# DECIDE for MOM Provider Training to improve healthcare providers’ competency in engaging and communicating with perinatal individuals: protocol for a non-randomized feasibility study

**DOI:** 10.1186/s40814-026-01857-z

**Published:** 2026-06-03

**Authors:** Christina D. Kang-Yi, Ora Nakash

**Affiliations:** 1 Department of Psychiatric Rehabilitation and Counseling Professions, School of Health Professions, Rutgers, the State University of New Jersey, Piscataway, NJ USA; 2 School of Social Work, Smith College, Northampton, MA USA

**Keywords:** Perinatal mental health, Shared decision making, Healthcare provider training, Adaptation of evidence-based practice, Dissemination and implementation, Health systems

## Abstract

**Background:**

Appropriate perinatal mental health care is an important public health concern as mental health disorders are common pregnancy complications. Perinatal and mental health care providers’ competencies in meeting mental health needs of perinatal individuals can increase access to appropriate mental health care. Despite the importance, perinatal and mental health care providers report discomfort in treating perinatal individuals with mental health needs due to the lack of evidence-based guidance for mental health treatment decisions, differences in beliefs and attitudes, and concerns about adverse effects on mental health and pregnancy outcomes. The proposed study aims to adapt the DECIDE provider training to improve care providers’ competencies in effectively engaging and communicating with perinatal individuals for shared decision making across pregnancy and postpartum stages in perinatal care settings. DECIDE stands for Decide the problem; Explore the questions; Closed or open-ended questions; Identify the who, why, or how of the problem; Direct questions to your health care professional; Enjoy a shared solution.

**Methods:**

The proposed study with a single-arm prospective design will make content adaptation (i.e., adding topical training content to fit perinatal mental health care) and process adaptation (i.e., creating asynchronous training modules to reduce the burden for care providers) to the DECIDE provider training. The acceptability, appropriateness, and usability of adapted DECIDE provider training for wide dissemination and rapid implementation in health systems will be assessed.

**Discussion:**

DECIDE teaches healthcare providers skills for perspective-taking, reducing attributional errors, and increasing receptivity to the client population. An adapted DECIDE provider training is expected to be an effective guiding tool for perinatal and mental health providers in delivering competent care for perinatal individuals with mental health disorders. This study will determine the acceptability, appropriateness, and usability of the adapted DECIDE provider training to support high quality perinatal mental health care delivery.

**Trial registration:**

ClinicalTrials.gov NCT07098260. Registered on 17 August 2025. https://clinicaltrials.gov/study/NCT07098260

## Introduction

Appropriate training for healthcare providers to meet unique mental health needs of perinatal individuals is an important public health concern. Mental health disorders among perinatal individuals are common pregnancy complications in the USA with 13% of perinatal individuals experiencing depression after childbirth and 43% of depressed perinatal individuals experiencing co-occurring anxiety [1]. Kang-Yi and colleagues’ research [2] found that over 80% and nearly 40% of low-income pregnant women with psychiatric disorders in one of the largest states in the USA drop out of psychotropic medication use and psychotherapy, respectively, early in their pregnancy. Despite the importance of appropriate perinatal mental health training, there is a lack of opportunities for structured training designed for healthcare providers such as psychiatrists, psychologists, counselors, psychiatric nurse practitioners, and other non-psychiatric providers such as midwives and obstetrics and gynecology clinicians who provide care for perinatal individuals with mental health needs [3–5]. These care providers report the difficulty of treating pregnant and postpartum women with mental health disorders due to the lack of evidence-based guidance for mental health treatment decisions during pregnancy, differences in beliefs and attitudes for diverse populations, and concerns about adverse effects on perinatal individuals such as self-harm and suicide [4, 6, 7].

Effective engagement and communication with perinatal individuals are essential competencies required for healthcare providers to successfully meet the unique mental health needs of perinatal individuals across pregnancy and postpartum stages in perinatal and mental health care settings [8, 9]. DECIDE is an evidence-based shared decision making intervention developed to improve shared decision making in behavioral health care through effective engagement and communication with patients with mental illness [10]. DECIDE stands for Decide the problem; Explore the questions; Closed or open-ended questions; Identify the who, why, or how of the problem; Direct questions to your health care professional; Enjoy a shared solution [10]. DECIDE provider training teaches healthcare providers skills for perspective-taking, reducing attributional errors, and increasing receptivity to the client population in shared decision making [10–13]. DECIDE including DECIDE-Patient Activation and DECIDE clinician intervention has demonstrated the effectiveness in improving patient activation [10, 14], patient self-management [12, 14], perceived quality of care [12], collaborative decision-making [10], and anxiety symptoms [13] when implemented in outpatient mental health care for adults. The DECIDE implementation in children’s behavioral healthcare setting has identified the need for adaptations of DECIDE to fit target populations and care settings [15].

## Methods

### Aim and objectives

This study aims to (1) make content adaptation (i.e., adding topical training content to fit perinatal mental health care) and process adaptation (i.e., creating asynchronous training modules to reduce the burden for care providers) to the DECIDE provider training and (2) assess the acceptability, appropriateness, and usability of adapted DECIDE provider training for wide dissemination and rapid implementation of DECIDE in maternal mental health care delivery. This study is approved by the Rutgers University Institutional Review Board.

### Study design

The study involves two phases.

#### Phase 1: Adapt DECIDE to translate the knowledge of DECIDE provider training into perinatal mental health care

The investigators will use Iterative Decision-Making for the Evaluation of Adaptations (IDEA) [16] and the Framework for Reporting Adaptations and Modifications-Enhanced (FRAME) [17] as guiding frameworks for the adaptation. Agency for Healthcare Research and Quality’s synthesis of research evidence for pharmacologic and nonpharmacologic treatments for maternal mental health care [18] will be used as the guiding tool for selecting the key questions for shared decision making. The example questions include the following: whether to offer nonpharmacologic interventions alone or in combination with pharmacologic intervention for specific perinatal mental health conditions and which nonpharmacologic interventions for perinatal mental health lead to optimal patient outcomes. The core content of the DECIDE provider training [10, 19] will be retained in a two-hour asynchronous training. The five modules include shared decision-making, perspective-taking (understanding perinatal individuals’ circumstances and perceptions), patient activation, attributional errors, and being a responsive provider to perinatal individuals. Clinical illustrations will be created to address patient and provider concerns for perinatal mental health, such as pharmacological mental health treatment in relation to breastfeeding, pregnancy complications, maternal harms and child outcomes, patient sociodemographic factors, mental illness and prior pregnancy history, and patient preferences.

*Stakeholder engagement to adapt DECIDE provider training*: The investigators will establish a provider advisory board composed of perinatal and mental health care providers and recruit subject matter experts with expertise in perinatal mental health care, shared decision making, and health systems.

*Recruitment of provider advisory board*: The investigators will recruit five provider advisory board members to guide adaptations to DECIDE provider training. The members will include those who provide perinatal mental health care such as nurses, psychologists, social workers, midwives, counselors, peer specialists, and other providers. The advisory board will help identify content adaptations to be made to fit mental health treatment for perinatal individuals, review asynchronous training modules and clinical illustrations, provide feedback on the provider training materials for revisions to be made, and advise how to best implement the adapted DECIDE provider training in health systems.

*Recruitment of subject matter experts*: The investigators will recruit three subject matter experts with expertise in perinatal mental health, physical and emotional well-being, symptom management, shared decision making, and health systems. The experts will guide key content adaptations to be made to the provider training modules, provide relevant resources to create the content adaptation, co-develop scripts for the training vignettes for clinical illustrations, and review provider training materials and make recommendations.

Focus groups will be conducted with the advisory board and subject matter experts to develop content adaptation and strategies for rapid implementation and wide dissemination of the DECIDE provider training in health systems. The focus groups will be held on a secure Health Insurance Portability and Accountability (HIPAA)-compliant Zoom. Focus groups through Zoom have been reported to be an excellent method for programming and managing focus groups [20, 21]. A semi-structured topic guide consisting of broad, open-ended questions will include questions for structured stakeholder consultation/discussion: (1) What are the anticipated key facilitators and barriers to using DECIDE as a shared decision making tool in health systems? (2) What are the components that must be included or avoided in the adaptations to the DECIDE provider training to ensure that perinatal and mental health providers in health systems use DECIDE skills taught? and (3) What are appropriate and acceptable strategies to integrate the adapted DECIDE provider training into routine perinatal care processes?

*Create, record, edit, and upload the adapted DECIDE provider training in Canvas*: Gathering stakeholders’ input, the investigators will adapt training content, develop scripts for vignettes, revise the training content and scripts through the iterative process of reviews and revisions by engaging the provider advisory board and the subject matter experts, and record training modules in Zoom webinars. Video clips for clinical illustrations created based on the scripts will be inserted into the adapted DECIDE provider training. This recorded asynchronous training will be edited and uploaded in a Canvas course.

#### Phase 2: Assess the acceptability, appropriateness, and usability of the adapted DECIDE provider training to build new knowledge of DECIDE implementation in perinatal mental health practice

The study will recruit 35 perinatal and mental health providers, including midwives, primary care doctors and nurses, social workers, psychologists, case managers, counselors, peer specialists, and obstetrics and gynecology care providers who provide mental health care to perinatal individuals within health systems. Health systems include hospitals and other healthcare organizations (e.g., home visit programs, state medical assistance and health services programs, federally qualified health centers).

##### Sample size justification

The primary aim of the study is to evaluate the feasibility and acceptability of adapted training, including recruitment, retention, training engagement, and data collection processes, rather than conduct hypothesis testing or establish efficacy. Following guidelines for sample sizes for feasibility studies [22, 23] for sufficient estimates of key feasibility parameters, the current study aims to recruit 35 participants, which is considered adequate for assessing feasibility outcomes and estimating variability in outcome measures.

Inclusion criteria:

- Perinatal or mental health care providers who currently provide perinatal mental health care within health systems
- Ages between 18 and 64
- Must be able to understand the training content in English and comfortably complete the assessments and share feedback in English

Exclusion criteria:

- Healthcare providers who do not practice as part of a health care system
- Healthcare providers who do not practice in the USA
- Healthcare providers who do not currently provide perinatal mental health care

The study will identify potential health systems that will implement adapted DECIDE provider training. Eligible participants in the health systems will receive an email invitation that includes participation information. Health system leaders reached out by the study team will help recruit by disseminating the training information and encouraging their clinicians to take the training. For participants interested in taking part, a study team member will email further information about the study, obtain electronic consent, collect baseline data, and offer instructions for taking the asynchronous online training.

### Intervention

Participants will take an asynchronous online DECIDE for MOM Provider Training, the adapted version of DECIDE provider training. The training will take approximately 2 h. The original core content of the DECIDE provider training [10], will be kept while clinical illustrations will be adapted to fit perinatal care settings. The five modules of the adapted DECIDE Provider Training will include shared decision-making, perspective-taking, patient activation, attributional errors, and being a responsive provider to perinatal individuals. Table 1 shows the participant enrollment, intervention and assessment timeline.

**Table 1:**
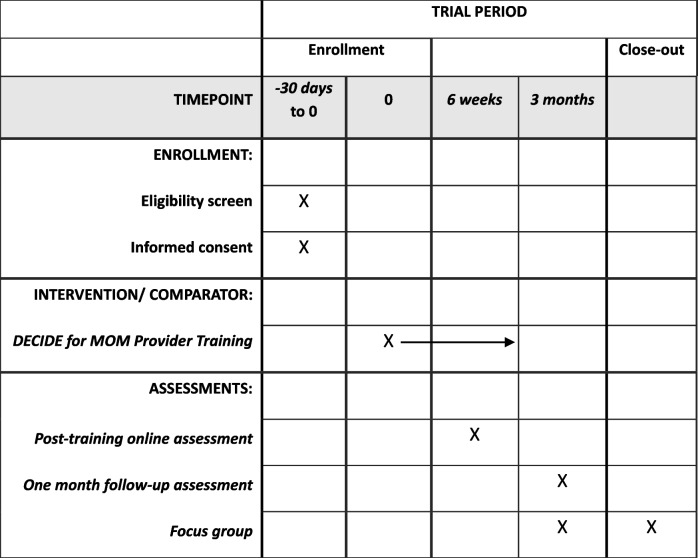
Participant Timeline: Schedule of Enrollment, Intervention, and Assessments

Note: SPRIT (Standard Protocol Items: Recommendations for Interventional Trials) template used for the report [24]

### Outcome measures

To identify the acceptability, appropriateness, and usability of adapted DECIDE provider training, the Acceptability, Feasibility, and Appropriateness Scale (AFAS) [25] developed for the assessment of implementation outcomes for training in evidence-based practices will be used. The measure has demonstrated a good fit to the hypothesized three-factor structures (RMSEA = 0.058, CFI = 0.990, TLI = 0.987) and acceptable internal consistency (α = 0.86 to 0.91). The AFAS uses a 5-point Likert scale with 1 indicating “not at all” and 5 indicating “extremely.” A few examples of the items are: “To what extent are you satisfied with the training you received and the practices covered?”, “How comfortable are you with the practices contained within the training?”, “How well do the information and practices fit with your overall approach to service delivery and the setting in which you provide care?” AFAS has demonstrated sensitivity to assessing workshop-specific perceptions about training acceptability and feasibility [25].

Open-ended questions will be added to obtain detailed information on user acceptability, appropriateness and usability. The example questions include the following: (1) Share your experience of taking the asynchronous online training regarding login, course navigation, module organization, and other aspects of accessing the training (2) Did the training change how you interact with perinatal individuals with mental health disorders? What are the specific examples? (3) What are the best strategies to effectively implement the DECIDE for MOM Provider Training within your organization?

The online assessment of acceptability, appropriateness, and usability will be conducted immediately after and one month after the training. Considering that immediately measuring acceptability, appropriateness, and usability can increase the correlation between appropriateness and usability but less highly correlated post-implementation of an intervention as providers perceive usability differently once they actually use the skills taught, [26] providers who complete the training will be asked to also participate in the one-month follow-up assessment.

Focus groups with the training participants will be conducted to gain an in-depth understanding of participants’ perceptions of acceptability, appropriateness, and usability of the adapted DECIDE provider training and thoughts about strategies to promote high-quality implementation of the training. The focus group meetings will be held on a secure HIPAA-compliant Zoom. The semi-structured topic guide will be used for structured stakeholder consultation/discussion: (1) What are the key facilitators and barriers to practicing DECIDE skills taught in perinatal mental health care? (2) What are the components that must be included or avoided in the DECIDE provider training to ensure that mental health providers in health systems use it as a shared decision making tool? and (3) What are appropriate and acceptable strategies to implement the DECIDE Provider Training as part of the routine perinatal mental health care at participants’ organization?

### Participant recruitment and retention

Thirty-five perinatal and mental health care providers who meet the inclusion and exclusion criteria will be recruited as participants for the training. A study team member will provide initial instructions for training course login and communicate with participants for technical issues via email. The study team will keep weekly participant log to support participant engagement and retention. Training and online assessment completion and focus group participation will be encouraged through email reminders. Participants will receive a $100 e-gift card for completing all five modules of the training and two online assessments and an additional $50 e-gift card for participating in a focus group and sharing in-depth feedback about the training, experience of using the DECIDE skills, and strategies for implementing the training as part of routine perinatal care in health systems.

### Criteria for feasibility

*Intervention uptake and engagement*: 80% or more registered participants logging in to the DECIDE for MOM Provider Training.

*Acceptability, Feasibility, and Appropriateness*: 3 (which indicates moderate agreement) or higher AFAS subscale mean scores for acceptability, feasibility and appropriateness at both immediately after the training and one-month follow-up assessments. Qualitative data collected through open-ended questions of the assessments and focus groups will be used to gain in-depth insights into perceived acceptability, appropriateness, usability including facilitators and barriers, and strategies for wide dissemination and rapid implementation of the adapted training in the real-world setting.

### Mixed-methods data analysis

All quantitative data will be collected and stored in a secure HIPAA-compliant Qualtrics database. All focus groups through Zoom will be transcribed through Zoom Caption and verified by the study team. Downloaded data for analyses will be stored in a HIPAA-compliant, password-protected Box folder. Descriptive statistics will be used to summarize quantitative data elements (e.g., provider demographics, years of experience in perinatal mental health care, number of months at the current job, and acceptability, appropriateness, and usability scores). Continuous variables will be summarized as means (standard deviation) if normally distributed, or median (range) for skewed data. Categorical data will be summarized as counts and percentages. Qualitative data from the focus groups will be analyzed using a deductive coding approach by creating pre-defined codes and then developing additional nodes from the participant recommendations. For the training participant data which will include both quantitative and qualitative data, the mixed-method data using a sequential structure, with an emphasis on the qualitative data (quanQUAL) will be used [27].

### Data monitoring

As this study is a small-scale feasibility study, no formal data monitoring committee will be convened. There is no harm anticipated as a result of taking part in this study other than discomfort. The online assessments will collect participant names and IP addresses. Upon downloading the assessment data, identifiers will be separated from the data. We will create IDs for participants to create a link to identifiers. The link will be stored in a HIPPA-compliant, password-protected Box folder. The Principal Investigator and the Research Assistants will have access to the data. Once the Zoom recordings are moved to the HIPPA-compliant Box folder, the study team will remove the recordings from Zoom. Identifying information linked to individual subject data will be removed after manuscripts on the study are published. The de-identified data will be maintained for six years after the completion of the study.

### Modification of the protocol

Modifications to the protocol will be approved by the Rutgers University Institutional Review Board.

## Discussion

American Psychiatric Association [5] emphasizes the importance of including perinatal psychiatry as the required competency for mental health professionals [28]. At present, the accreditation and training requirements across mental health disciplines, including psychiatry, psychology, counseling, social work, and nursing, do not mandate training on perinatal mental health [5]. Adapted DECIDE for MOM Provider Training is expected to be an effective guiding tool for mental health providers in meeting the unique mental health needs of perinatal individuals. Given that primary care and obstetrics and gynecology care providers are often the first point of contact for perinatal individuals, widely disseminating the DECIDE for MOM Provider Training can play an important role in improving competencies of all perinatal care providers in meeting the mental health needs of perinatal individuals.

DECIDE provider training is evidence-based and teaches skills for perspective-taking, reducing attributional errors, and increasing receptivity to the client population in shared decision making. This mixed-methods feasibility study will be the first study that determines the acceptability, appropriateness and usability of the adapted DECIDE provider training to support high quality perinatal mental health care delivery in health systems. The findings will inform the future large-scale evaluation of DECIDE for MOM if found to be appropriate. Study results will be reported through publications, presentations, and ClinicalTrials.gov to communicate results to interested parties such as participants, healthcare professionals, and the public.

## Data Availability

Not applicable. This manuscript does not include any data.

## Abbreviations

DECIDE: Decide the problem; Explore the questions; Closed or open-ended questions; Identify the who, why, or how of the problem; Direct questions to your health care professional; Enjoy a shared solution
HIPPA: The Health Insurance Portability and Accountability
